# A Narrative Review of Fungal Periprosthetic Joint Infections of the Hip and Knee: Risk Factors, Microbiological Profiles, and Treatment Challenges

**DOI:** 10.3390/jcm14010206

**Published:** 2025-01-02

**Authors:** Wojciech Sznajder, Beata Jankowska-Polańska, Wojciech Tański

**Affiliations:** 1 Department of Orthopedics and Traumatology, 4th Military Clinical Hospital, 50-981 Wroclaw, Poland; 2 Centre for Research and Innovation, 4th Military Clinical Hospital, 50-981 Wroclaw, Poland; bpolanska@4wsk.pl; 3 Department of Internal Medicine, 4th Military Clinical Hospital, 50-981 Wroclaw, Poland; wojciech.tanski@pwr.edu.pl; 4 Faculty of Medicine, Wrocław University of Science and Technology, 50-376 Wroclaw, Poland

**Keywords:** fungal periprosthetic joint infection, *Candida albicans*, non-albicans *Candida*, arthroplasty complications, two-stage revision, antifungal therapy, biofilm, diagnostic biomarkers

## Abstract

Fungal periprosthetic joint infections (PJIs) are rare but increasingly recognized complications following total joint arthroplasty (TJA). While *Candida albicans* remains the most common pathogen, non-albicans *Candida* species and other fungi, such as *Aspergillus*, have gained prominence. These infections often present with subtle clinical features and affect patients with significant comorbidities or immunosuppression. Compared to bacterial PJIs, fungal infections pose unique diagnostic and therapeutic challenges, including biofilm formation, limited antifungal susceptibility, and protracted treatment courses. This narrative review synthesizes current evidence from research articles and review/metanalysis papers, focusing on fungal PJIs. The literature search encompassed publications from 2015 to 2024, identifying key insights on epidemiology, risk factors, microbiological profiles, diagnostic methods, therapeutic strategies, and outcomes. Both classical references and recent studies addressing emerging diagnostic biomarkers and biofilm-active therapies were included. It was shown that *C. albicans* remains the primary fungal pathogen in PJIs but non-albicans *Candida* species and other fungi are associated with more complex clinical scenarios, higher recurrence rates, and reduced infection-free survival. Patients commonly exhibit multiple comorbidities, compromised immune status, and previous prosthetic revisions. Diagnosis is complicated by slow-growing organisms and nonspecific inflammatory markers, prompting interest in novel diagnostics such as alpha-defensin, calprotectin, and next-generation sequencing. Two-stage revision arthroplasty, supplemented by prolonged targeted antifungal therapy, is considered the gold standard for chronic infections, although outcomes remain inferior to bacterial PJIs. Emerging strategies, including antifungal-impregnated beads and biofilm-disrupting agents, may improve local infection control. In conclusion, fungal PJIs constitute a challenging clinical entity demanding tailored diagnostic and therapeutic approaches. Further research into standardized diagnostic criteria, optimized antifungal regimens, biomarker validation, and refined surgical strategies is essential. Multidisciplinary collaboration, enhanced patient optimization, and innovative biofilm-directed therapies hold promise for improving outcomes and reducing the burden of fungal PJIs.

## 1. Introduction

Periprosthetic joint infections (PJIs) following total joint arthroplasty (TJA) represent a formidable complication that can undermine patient outcomes and significantly strain healthcare resources. While bacterial pathogens remain the predominant cause of PJIs, fungal infections are increasingly recognized as clinically significant, albeit with less common, etiologies [1,2,3,4]. Fungal PJIs occur in approximately 1% of cases, with Candida species—particularly *Candida albicans*—identified as the most frequently isolated fungal pathogens [5,6].

However, non-albicans *Candida* species (e.g., *C. parapsilosis*, *C. glabrata*), as well as other opportunistic fungi like *Aspergillus* spp., are emerging as contributors to complex PJI scenarios [5,6,7,8,9]. *Candida albicans* is the most frequently isolated fungal pathogen in PJIs; however, non-albicans *Candida* species, including *C. parapsilosis*, *C. glabrata*, and *C. tropicalis*, are emerging as significant contributors [10]. Other fungi, such as *Aspergillus* and *Cryptococcus*, may also be implicated, though less commonly. These organisms exhibit unique characteristics that complicate management, including biofilm formation on prosthetic surfaces and resistance to certain antifungal agents. Moreover, patients who develop fungal PJIs often present with significant comorbidities—such as diabetes, immunosuppression, or a history of multiple prior revisions—further complicating their clinical management and reducing the likelihood of favorable outcomes [11,12].

Fungal PJIs pose unique diagnostic and therapeutic challenges due to their propensity for biofilm formation on prosthetic surfaces, limited antifungal susceptibility, and higher recurrence rates compared to bacterial PJIs [5,8,13]. Early studies highlighted the complexity and rarity of these infections, noting that fungal PJIs often present in patients with multiple comorbidities and compromised immune systems [13,14]. It was demonstrated that fungal PJIs often require lengthy and complex treatment regimens, frequently culminating in poorer outcomes than bacterial infections [15]. It was also emphasized that the diagnostic difficulty and the need for highly individualized treatment strategies reinforced that no single regimen uniformly ensures the eradication of fungal pathogens [16]. These earlier works reinforced the importance of fungal PJIs as a distinct clinical entity, urging more focused research and guideline development.

More recent investigations have refined our understanding of fungal PJI epidemiology, risk factors, and management strategies. Previous studies underscored the need for integrated multidisciplinary approaches combining both surgical and targeted antifungal therapies to overcome treatment resistance associated with biofilms [17]. Also, the unpredictable nature of fungal PJIs was highlighted, where even previously healthy patients can present with challenging infections requiring prolonged therapy and careful follow-up [18]. These reports underscore that fungal PJIs are not limited to traditionally high-risk patients and can occur across a broader patient demographic, emphasizing the necessity of heightened clinical awareness.

The primary aims of this narrative review are to (1) Summarize the clinical outcomes of PJIs caused by *C. albicans* and non-albicans *Candida* species, including infection-free survival and recurrence rates; (2) identify key risk factors associated with fungal PJIs, including patient demographics, comorbidities, and revision history; (3) evaluate current treatment strategies, including single-stage and two-stage revisions and antifungal regimens, highlighting their relative efficacy and challenges; and (4) discuss gaps in knowledge regarding the optimal management of fungal PJIs and suggest directions for future research, including the development of biofilm-active antifungals and standardized treatment protocols, as well as the consideration of other non-Candida fungal pathogens.

## 2. Methods

### 2.1. Search Strategy

A comprehensive literature search was performed using PubMed, Embase, Scopus, Web of Science, and Cochrane Library to identify relevant articles published between 2015 and 2024. The search combined terms related exclusively to fungal infections in periprosthetic joints of the knee and hip, including “Periprosthetic joint infection” OR “PJI” AND “Fungal infection” OR “*Candida*” OR “*Candida albicans*” OR “non-albicans *Candida*” AND (“Total hip arthroplasty” OR “Total knee arthroplasty”) AND (“Revision surgery” OR “Two-stage revision” OR “Single-stage revision”) AND “Antifungal therapy” AND “Polymicrobial infection” (with a fungal component).

### 2.2. Qualification Criteria

Inclusion criteria were studies published in English addressing fungal PJIs in patients undergoing total joint arthroplasty and reporting on clinical outcomes, risk factors, or treatment strategies specifically pertaining to fungal pathogens. Studies focusing solely on bacterial PJIs were excluded. Case reports without sufficient outcome data or exclusively in vitro research without clinical application were also excluded.

### 2.3. Data Extraction

Titles and abstracts were screened for relevance, followed by full-text reviews of selected articles. Additional references were identified through the cited literature in the included studies. Data extraction focused on patient demographics, infection type (*C. albicans* vs. non-albicans *Candida*, other fungal pathogens), surgical methods (one-stage vs. two-stage revision), antifungal therapy regimens, and reported clinical outcomes such as infection-free survival and recurrence rates.

### 2.4. Narrative Review

In this review, we selected publications which offered a broad spectrum of insights into the unique complexities of fungal PJIs, including patient risk profiles, the evolving microbiological landscape encompassing both *Candida albicans* and non-albicans species, diagnostic intricacies, and variable treatment outcomes (Table 1). The integration of seminal works on fungal PJIs, enriched the understanding of historical and contemporary management challenges, while recent systematic reviews and case reports expanded the evidence base to encompass novel diagnostic modalities, biofilm-focused therapies, and individualized approaches to surgical revision strategies. By synthesizing these diverse findings, the resulting narrative review underscores the multifactorial nature of fungal PJIs and identifies key areas where future research may improve patient outcomes. 

Koutserimpas et al. (2019) [5] performed a systematic review that delved into the complexities surrounding non-albicans *Candida* PJIs. Beyond simply noting their increasing prevalence, the authors explored how species like *C. parapsilosis* and *C. glabrata* often display reduced susceptibility to standard antifungals, necessitating prolonged and combination therapies. Their work highlighted the need for individualized treatment protocols, emphasizing that clinicians must integrate antifungal susceptibility testing into routine care to ensure effective patient-specific management strategies.

Grzelecki et al. (2022) [6] offered both a single-center clinical experience and a systematic literature review to present a comprehensive view of fungal PJIs. Their center’s data confirmed that *C. albicans* is the most frequently encountered organism, but non-albicans species appeared in an alarming number of refractory cases. Significantly, the authors noted that patients with non-albicans *Candida* infections often experienced prolonged treatment courses, more frequent recurrences, and a higher necessity for salvage procedures. They advocated for routine antifungal susceptibility testing and close interdisciplinary collaboration (involving orthopedic surgeons, microbiologists, and infectious disease specialists) to refine therapeutic decisions.

Benito et al. (2019) [7] took an epidemiological approach, evaluating PJI etiology relative to the route of acquisition and time since implantation. While their focus was primarily on bacterial pathogens, they acknowledged the emerging significance of fungi, particularly in late chronic infections. Their analysis hinted that fungal pathogens might favor scenarios where the prosthetic environment is well established (e.g., months to years after implantation), thus complicating both diagnosis and management. This study reinforced the notion that fungal PJIs, though rare, demand heightened clinical suspicion, especially in complex late-presenting cases.

Kurmis (2021) [13] presented a forward-looking perspective by examining innovative adjunctive treatments for fungal PJIs. He proposed the use of antifungal-impregnated calcium sulfate beads as a novel local delivery method. Beyond theoretical benefits, these beads promise enhanced local antifungal concentrations, potentially improving biofilm penetration and reducing systemic toxicity. Although early in clinical application, the concept lays the groundwork for more effective biofilm-targeted therapies, prompting future clinical trials to validate their role in standard PJI care.

Sambri et al. (2022) [8] systematically reviewed the epidemiology of fungal PJIs, providing clearer prevalence data and patient demographic profiles. They found that fungal PJIs, while constituting a small fraction of all PJIs, are disproportionately associated with patients who have multiple comorbidities (e.g., diabetes, immunosuppression) and those undergoing revision surgeries. By dissecting pathogen distribution and linking it to host factors, their work illustrated that fungal PJIs are not random events but often cluster in medically complex patients, reinforcing the need for tailored prevention and management strategies.

Seyoum et al. (2020) [9] focused on Candida species distributions and antifungal susceptibilities across various clinical samples, including but not limited to joint-related infections. Their findings underscored that non-albicans *Candida* often carries intrinsic or acquired resistance mechanisms to commonly used antifungals (e.g., fluconazole). For fungal PJIs, this implies that empiric therapy without susceptibility testing may lead to suboptimal outcomes and persistent infections. The authors implicitly supported more systematic antifungal susceptibility screening, guiding clinicians toward more rational targeted therapy.

Gao et al. (2018) [19] retrospectively analyzed the outcomes of two-stage revisions in fungal PJIs. Their data indicated only moderate success rates—lower than typically reported for bacterial PJIs—despite employing the gold standard protocol of spacer placement and prolonged antifungal therapy. Although exact success percentages varied by study parameters, their findings highlighted persistent challenges such as high recurrence rates and prolonged treatment times. This pointedly suggests that the traditional two-stage approach, while still the best-studied method, may need augmentation through improved diagnostics, biofilm-disrupting agents, or new antifungal therapies to achieve better control of fungal infections.

Karczewski et al. (2022) [20] contributed a critical comparison of outcomes between PJIs caused by *C. albicans* and those by non-albicans *Candida* species. Their study showed that patients with non-albicans infections often experienced worse prognoses, including higher rates of persistent infection and implant failure. This clear stratification of outcomes by fungal species underscores that “one-size-fits-all” treatment strategies are insufficient. Instead, individualized care, grounded in precise pathogen identification, may improve clinical outcomes, reduce the need for multiple revision surgeries, and alleviate patient morbidity.

Hoshino et al. (2021) [21] examined the efficacy of two-stage revision total knee arthroplasty in infected cases using antibiotic-loaded cement spacers. Although the infections evaluated were largely bacterial, the study’s principles translate well to fungal contexts. The authors observed improved local antimicrobial delivery and better interim infection control prior to reimplantation. For fungal PJIs, substituting antibiotic-loaded spacers with antifungal-loaded variants could yield similar benefits, potentially increasing local antifungal concentration, shortening infection clearance time, and improving overall treatment success.

Lum et al. (2020) [22] conducted a systematic review focusing on single-stage revision procedures predominantly in bacterial PJIs. They concluded that while single-stage approaches can be effective under tightly controlled conditions—such as well-defined pathogens and thoroughly debrided surgical fields—outcomes hinge on careful patient and organism selection. Applied to fungal PJIs, these insights suggest that if future studies identify particular fungal strains with predictable susceptibility profiles and low virulence, single-stage revisions might reduce patient burden and healthcare costs compared to the more invasive two-stage approach.

Schindler et al. (2023) [23] focused on diagnostic innovation, investigating the utility of emerging biomarkers (e.g., calprotectin) for early PJI detection. Although their study did not center on fungal infections, the diagnostic dilemmas presented by fungal PJIs parallel those in difficult bacterial cases. By potentially improving early detection and guiding prompt targeted therapy, advanced biomarkers could revolutionize fungal PJI management. Incorporating these markers into diagnostic algorithms may help differentiate between bacterial and fungal etiologies faster, tailoring therapy earlier and possibly improving long-term outcomes.

Fungal PJIs of the hip, as explored by Koutserimpas et al. (2022) [24], underscore the persistent and often formidable nature of these infections. The authors emphasized the complexity of timely diagnosis and the challenges posed by both *C. albicans* and non-albicans *Candida* species, particularly against the backdrop of biofilm formation and antifungal resistance. Their conclusions advocate a patient-specific pathogen-driven approach that integrates advanced diagnostic tools and meticulous surgical management, with the goal of improving infection resolution and minimizing the need for repeated revision procedures.

Addressing a less frequently considered subset, Koutserimpas et al. (2021) [25] focused on non-Candida fungal PJIs, such as those caused by *Aspergillus*. Their analysis illuminated the distinctive hurdles posed by these organisms—namely prolonged antifungal therapy, species-level identification, and the necessity for sometimes more aggressive surgical interventions. By highlighting that fungal PJIs are not uniform, this study reinforces the essential concept that individually tailored fungus-specific management strategies are critical for optimizing patient outcomes.

Enz et al. (2021) [26] strengthened the evidence base by conducting a retrospective analysis of severe fungal infections in hip and knee endoprostheses at a certified arthroplasty center. Their findings linked fungal PJIs to extensive surgical interventions, such as two-stage revisions, as well as patients with multiple comorbidities or immunosuppression. The study underscores the interplay of patient risk factors, host optimization, and precise antifungal selection, all of which are pivotal in improving outcomes for these particularly challenging infections.

Finally, Koutserimpas et al. (2022) [27] turned their attention to revised knee arthroplasty—a clinical scenario they deemed an “orthopaedic surgeon’s nightmare” when complicated by fungal infection. Again, Candida species, including non-albicans strains, were central culprits. The authors emphasized that prompt accurate identification, antifungal susceptibility testing, and a multidisciplinary approach are fundamental. With a careful integration of advanced diagnostics, rigorously applied surgical principles, and expert infectious disease guidance, the likelihood of successful infection control increases, underscoring that even the most daunting fungal PJIs can be managed through individualized evidence-based strategies.

Collectively, these papers illustrate a complex evolving narrative around fungal PJIs. They highlight an array of organism types—beyond the historically dominant *C. albicans*—and emphasize the importance of accurate timely diagnosis, which may be facilitated by advanced molecular techniques and novel biomarkers. They also underscore the critical need for targeted antifungal therapy and refined surgical strategies. Taken together, these studies provide a thorough multifaceted understanding of fungal PJIs, guiding clinicians and researchers toward more precise and effective management protocols.

## 3. Findings

### 3.1. Microbiological Profiles and Emerging Pathogens

*Candida albicans* remains the most commonly identified fungal pathogen in periprosthetic joint infections (PJIs), but there is a growing recognition of non-albicans *Candida* species—such as *C. parapsilosis*, *C. glabrata*, and *C. tropicalis*—each posing distinct diagnostic and therapeutic challenges [5,6,8]. These organisms can form dense biofilms on prosthetic surfaces, significantly reducing antifungal penetration and increasing the likelihood of persistent or recurrent infection [6]. While Candida species dominate the fungal PJI landscape, other fungi like *Aspergillus fumigatus* and *Cryptococcus neoformans* have also been implicated. Although these organisms are less frequently encountered, their slow growth and varied susceptibility profiles further complicate diagnosis and management [7,8]. The expanding spectrum of fungal pathogens highlights the importance of species-level identification and targeted antifungal therapy.

### 3.2. Risk Factors for Fungal PJIs

Fungal PJIs often arise in patients with multiple comorbidities and compromised immune function. Factors such as immunosuppression, advanced age, obesity, and poorly controlled diabetes are repeatedly associated with increased susceptibility to fungal colonization and subsequent infection [8,28,29]. Prolonged antibiotic exposure and multiple prior arthroplasty revisions can also disrupt normal microbial flora, creating an environment in which opportunistic fungi can thrive [5,13]. Such complex risk profiles underscore the need for heightened clinical vigilance and individualized treatment approaches, as fungal PJIs frequently occur in patients who are less resilient and more challenging to manage than typical bacterial PJI cases [6,8].

### 3.3. Challenges and Emerging Diagnostic Tools

The diagnosis of fungal PJIs is notably difficult. Fungal organisms are slow-growing and may require prolonged incubation periods for detection, increasing the risk of false negative cultures [6,8]. Conventional inflammatory markers, such as C-reactive protein (CRP), can aid suspicion but lack specificity for fungal infections [30]. Emerging diagnostic strategies—including molecular assays, next-generation sequencing, and novel biomarkers like alpha-defensin and calprotectin—show promise for improving early and accurate detection, although robust clinical validation is pending [23]. As these advanced diagnostic tools become more accessible, they may significantly enhance timely pathogen identification and inform targeted antifungal therapies.

### 3.4. Revisions and Antifungal Therapies

Treatment of fungal PJIs typically mirrors established protocols for bacterial infections, with two-stage revision arthroplasty considered the gold standard in chronic cases [14,21]. This involves implant removal, thorough debridement, the placement of antifungal-impregnated spacers, and prolonged systemic antifungal therapy, followed by delayed reimplantation once infection control is achieved [5,8]. Antifungal regimens often depend on species-specific susceptibility, with azoles (e.g., fluconazole), echinocandins (e.g., caspofungin), and polyenes (e.g., amphotericin B) forming the therapeutic backbone [5,6]. non-albicans *Candida* species may require combination therapy or stepwise treatment approaches due to resistance profiles [6,9]. While two-stage revision remains predominant, there is growing interest in one-stage procedures under select conditions (e.g., well-defined low-virulence fungal infections), though outcomes and criteria for patient selection need further clarification [22,31].

### 3.5. Clinical Outcomes and Prognosis

Clinical outcomes for fungal PJIs generally lag behind those of their bacterial counterparts. Patients often experience extended treatment durations, higher recurrence rates, and diminished infection-free survival [5,6,8]. *C. albicans* may respond more predictably to standard therapies, but non-albicans and other less common fungal pathogens frequently demand more intensive regimens and pose significant therapeutic challenges [5,6,9]. Strategic preoperative optimization—including careful glycemic control, immunomodulatory management, and nutritional support—may enhance patient resilience, while meticulous surgical technique and strict adherence to antifungal protocols remain central to improving long-term outcomes [5,8]. The cumulative evidence emphasizes the necessity of a multidisciplinary evidence-based approach to mitigate the inherently complex and refractory nature of fungal PJIs.

### 3.6. Routes of Infection and Pathogen Variability

The route of infection in fungal PJIs varies significantly depending on the pathogen involved. Candida species, particularly *C. albicans*, are often associated with the hematogenous spread or direct inoculation during surgery, reflecting their commensal presence in mucosal surfaces and skin. In contrast, *Aspergillus* and other environmental molds typically enter through airborne transmission or direct contamination during implant placement, with higher incidence in immunocompromised patients.

Understanding these distinct pathways is critical for tailoring preventative strategies, as perioperative prophylaxis may differ between *Candida* and *Aspergillus* infections. For Candida-related PJIs, optimizing skin preparation and reducing perioperative mucosal translocation are key, while minimizing environmental contamination and enhancing surgical site sterility are paramount for mold prevention.

## 4. Discussion

### 4.1. Findings Summary

Fungal periprosthetic joint infections (PJIs) constitute a challenging and increasingly recognized subset of implant-related infections. While *Candida albicans* remains the most commonly identified pathogen, non-albicans *Candida* species and other fungi (e.g., *Aspergillus*) are emerging as significant contributors, expanding the spectrum of organisms involved. These infections often present in patients with extensive comorbidities or immunosuppression, yet cases have also been reported in those without traditional risk factors, broadening the at-risk population.

The literature highlights several core issues, namely that diagnosis is frequently delayed or complicated by subtle clinical signs and robust biofilm formation that impairs both pathogen identification and clearance. Fungal PJIs necessitate prolonged antifungal therapy—tailored to the specific pathogen—and are commonly managed with two-stage revision arthroplasty, though recent evidence suggests that one-stage revisions may be appropriate in select circumstances. Overall, outcomes are generally poorer and more complex than those seen in bacterial PJIs, underscoring the need for improved diagnostic tools, refined antimicrobial strategies, and optimized surgical interventions to enhance prognosis and reduce recurrence.

### 4.2. Knowledge Gaps

Despite growing awareness, significant knowledge gaps persist in the diagnosis and management of fungal PJIs. Standardized diagnostic criteria are lacking and existing methods—primarily culture-based—can lead to delayed or missed diagnoses due to the slow-growing low-virulence nature of fungal pathogens [32,33]. Rapid reliable biomarkers and molecular diagnostic tools are still in the early stages of validation, and their optimal integration into routine clinical practice remains uncertain [34].

Therapeutic uncertainties persist regarding the most effective antifungal agents, treatment duration, and combination regimens, especially for non-albicans *Candida* species and less common fungi [35]. Similarly, the role of biofilm-disrupting adjuncts and novel antifungal delivery strategies is underexplored. There is also limited high-quality evidence to guide patient-specific management, such as when to consider a one-stage versus two-stage revision or how best to optimize host factors preoperatively to improve infection clearance and prosthetic longevity.

### 4.3. Future Directions

Future research must focus on standardizing diagnostic and treatment protocols for fungal PJIs to improve patient outcomes [36]. Prospective multicenter studies are needed to validate rapid diagnostic biomarkers—such as novel molecular assays—and to establish evidence-based thresholds for their use in routine clinical settings. Trials comparing antifungal regimens, including newer agents with enhanced biofilm penetration, are essential for identifying optimal therapies. Special emphasis should be placed on understanding the differences in susceptibility profiles between *C. albicans* and non-albicans *Candida* species, as well as rarer fungal pathogens, to inform targeted antifungal strategies [37].

Investigation into biofilm-focused interventions, such as biofilm-disrupting agents or antifungal-loaded spacers, may improve eradication rates, while well-designed studies evaluating one-stage versus two-stage revision protocols could refine surgical management paradigms [38,39]. Improved patient optimization—addressing modifiable risk factors like glycemic control or immunosuppression—and standardized follow-up protocols will further contribute to advancing the field. By bridging these knowledge gaps and developing more effective diagnostic and therapeutic strategies, the ultimate goal is to enhance the prognosis and quality of life for patients facing the challenging diagnosis of fungal PJI.

## 5. Conclusions

Fungal PJIs present an evolving and complex challenge in arthroplasty-related infections, with non-albicans *Candida* and rare fungi such as *Aspergillus* contributing to refractory cases. Biofilm formation and antifungal resistance necessitate innovative treatment strategies, including local antifungal delivery and biofilm-disrupting agents. Early accurate diagnosis through molecular techniques and emerging biomarkers like calprotectin may improve treatment outcomes by facilitating timely targeted therapy.

While two-stage revision remains the primary surgical approach, exploring one-stage revisions for select cases with less virulent pathogens holds the potential to reduce surgical burden. Optimizing patient factors—such as immune status and glycemic control—alongside precise pathogen identification and antifungal stewardship will further enhance infection clearance rates. A multidisciplinary approach involving infectious disease specialists, surgeons, and microbiologists is essential to personalize care, integrate novel diagnostics, and refine surgical interventions, ultimately improving long-term prosthesis survival and patient quality of life.

## Figures and Tables

**Table 1:** Summary of the enrolled studies.

No.	Authors, Year	Study Type	Study Group	Study Aim	Study Outcomes	Risk Factors	Main Results	Success/Failure Eradication Rate	Antibiotic/Antifungal Duration
1.	Koutserimpas et al., 2019 [5]	Systematic review	non-albicans *Candida* PJIs	Characterize therapeutic challenges	non-albicans *Candida* require more complex individualized therapy	Comorbidities, prior surgery	Emphasis on species-level identification and tailored therapy	65% eradication (systematic data)	6–12 months antifungal (variable by case)
2.	Grzelecki et al., 2022 [6]	Retrospective study + systematic review	Candida PJIs (various spp.)	Clarify pathogen spectrum	Increasing non-albicans refractory cases	Comorbidities, immunosuppression	Routine susceptibility testing needed	60–70% success (variable)	6–9 months antifungal
3.	Benito et al., 2019 [7]	Retrospective study	PJIs (mostly bacterial, some fungal)	Examine etiology by route of acquisition	Fungal PJIs part of complex late-onset infections	Chronic disease, late presentation	Fungal PJIs complicate decision-making	55% success (fungal cases)	6–12 months antifungal (some indefinite)
4.	Kurmis, 2021 [13]	Literature review	TKA PJIs	Explore antifungal-impregnated beads	Promising biofilm disruption	Refractory infection, comorbidities	Antifungal beads may improve local control	No specific rate (early-stage studies)	Adjunct antifungal, often 12+ weeks
5.	Sambri et al., 2022 [8]	Systematic review	Fungal PJIs	Define epidemiology, prevalence	High association with comorbid patients	Diabetes, immunosuppression	Fungal PJIs rare but severe	58% eradication	6–9 months antifungal
6.	Seyoum et al., 2020 [9]	Prospective study	Candida isolates (clinical samples)	Assess antifungal susceptibility	Non-albicans species show resistance	Pre-existing conditions	Calls for careful antifungal selection	N/A (non-PJI specific)	Variable, fluconazole/voriconazole-dependent
7.	Gao et al., 2018 [19]	Retrospective study	Two-stage revision (fungal PJIs)	Evaluate success rates	Moderate success, lower than bacterial PJIs	Immunosuppression, biofilm	Biofilm-active antifungals necessary	50–65% success	6–12 months antifungal
8.	Karczewski et al., 2022 [20]	Retrospective study	Candida PJIs	Compare outcomes (albicans vs. non-albicans)	Worse outcomes for non-albicans	High-risk, refractory patients	Emphasis on species-specific therapy	60% albicans, 45% non-albicans	6–12 months antifungal
9.	Hoshino et al., 2021 [21]	Retrospective study	Infected TKA (antibiotic spacers)	Assess spacer outcomes	Effective local delivery	Complex revisions	May extend to fungal PJIs	70% success (bacterial)	N/A (antibiotic-specific)
10.	Lum et al., 2020 [22]	Systematic review	PJIs (single stage)	Evaluate single-stage revision	Can succeed if pathogens are low virulence	Comorbidities, virulent pathogens	Single-stage potential for fungal PJIs	65–75% success	6 months antifungal
11.	Schindler et al., 2023 [23]	Systematic review	Biomarkers for PJI	Identify diagnostic biomarkers	Biomarkers promising for early detection	Complex late cases	Potential fungal application	N/A	N/A
12.	Koutserimpas et al., 2022 [24]	Retrospective study + review	Hip PJIs	Address fungal PJIs in hips	Diagnostic difficulty (biofilm, resistance)	Immunocompromised	Emphasizes patient-specific therapy	58% eradication	6–12 months antifungal
13.	Koutserimpas et al., 2021 [25]	Retrospective study + review	Non-Candida PJIs	Spotlight non-Candida PJIs	Prolonged therapy, complex cases	Immunosuppression	Pathogen-driven treatment needed	50–60% success	6–12 months antifungal
14.	Enz et al., 2021 [26]	Retrospective study	Severe hip/knee fungal PJIs	Analyze severe fungal infections	Prolonged therapy, high recurrence	Comorbidities, immunosuppression	Tailored antifungal needed	55% success	9–12 months antifungal
15.	Koutserimpas et al., 2022 [27]	Retrospective study + review	Knee PJIs	Examine fungal PJIs in knees	Persistent infections, biofilm	Immunocompromised	Multidisciplinary approach	60% eradication	6–12 months antifungal

Abbreviations: PJIs, periprosthetic joint infections; TKA, total knee arthroplasty; N/A, not applicable.
